# RAiSD detects positive selection based on multiple signatures of a selective sweep and SNP vectors

**DOI:** 10.1038/s42003-018-0085-8

**Published:** 2018-06-27

**Authors:** Nikolaos Alachiotis, Pavlos Pavlidis

**Affiliations:** Institute of Computer Science, Foundation for Research and Technology-Hellas, Nikolaou Plastira 100, 70013 Heraklion, Crete Greece

## Abstract

Selective sweeps leave distinct signatures locally in genomes, enabling the detection of loci that have undergone recent positive selection. Multiple signatures of a selective sweep are known, yet each neutrality test only identifies a single signature. We present RAiSD (Raised Accuracy in Sweep Detection), an open-source software that implements a novel, to our knowledge, and parameter-free detection mechanism that relies on multiple signatures of a selective sweep via the enumeration of SNP vectors. RAiSD achieves higher sensitivity and accuracy than the current state of the art, while the computational complexity is greatly reduced, allowing up to 1000 times faster processing than widely used tools, and negligible memory requirements.

## Abstract (web summary)

Nikolaos Alachiotis and Pavlos Pavlidis present RAiSD, a computational method for identifying multiple signatures of selective sweeps using single nucleotide polymorphism vectors. They show that RAiSD has higher sensitivity and accuracy with reduced computational complexity than current methods.

## Introduction

Positive selection is the major driving force behind adaptation^1^. Its detection has practical applications such as identifying drug-resistant mutations in pathogens^2^ and designing more effective drug treatments^3^. Smith and Haigh^4^ described the effect of genetic hitchhiking as the change in frequency of neutral alleles at loci that are linked to a selected locus. The beneficial allele at the selected locus increases in frequency while linked neutral variation diminishes, creating a so-called selective sweep. Detecting selective sweeps is feasible due to three distinct signatures that a sweep leaves in genomes. The first signature is the local reduction of the polymorphism level^4^. The second signature suggests a particular shift in the site frequency spectrum (SFS) toward low- and high-frequency derived variants^5^. Finally, the third signature is a localized pattern of linkage disequilibrium (LD) levels^6^, characterized by high LD on each side of a beneficial mutation and low LD between loci that are located on different sides of the beneficial allele.

The currently available methods for selective sweep detection exhibit various disadvantages. First, each method is designed to only detect one of the aforementioned sweep signatures, and a common practice to increase the credibility of an analysis is to examine the outcome of multiple neutrality tests. This approach, however, may be suboptimal, as the results of some neutrality tests may be correlated to a certain extent since they depend on the same underlying coalescent trees^6^, or lead to conflicting outcomes. Second, state-of-the-art detection methods, such as SweepFinder^7^, SweepFinder2^8^, SweeD^9^, and OmegaPlus^10^, require input parameters that determine how exhaustively each tool is going to scan a dataset. The aforementioned implementations construct a grid of equidistant locations to evaluate, having implications for the accuracy of the detection process and the computational efficiency of the applied methods. The user’s choice of the grid size may lead to execution scenarios where no grid point is placed in the region of a selected locus, while the placement of grid points along a genome based on the size of the evaluated genomic region in base-pairs (bp) may lead to redundant calculations in regions with a reduced number of single nucleotide polymorphisms (SNPs) (Supplementary Note 1). Third, software tools exhibit increased computational and/or memory requirements, even when processing rather small datasets. SweepFinder2 required more than 5 h to scan 1000 simulated datasets with 20 sequences and 2215 SNPs on average, evaluating 1000 grid points, while OmegaPlus failed to process the first chromosome of the human genome (data available from the 1000 Genomes project^11^) on a high-end, off-the-shelf personal computer with 32 GB of main memory, due to excessive memory requirements for parsing the input file (65.8 GB). Given the continuous DNA-sequencing efforts worldwide, software tools that are incapable of rapidly analyzing large-scale datasets with reasonable memory requirements and, more importantly, without compromising the accuracy and the sensitivity of the detection process will soon be deemed unsuitable for sweep scans on personal computers.

To this end, we introduce the *μ statistic*, a composite evaluation test that scores genomic regions by quantifying changes in the SFS, the levels of LD, and the amount of genetic diversity along a chromosome. Despite the fact that the *μ statistic* relies on three sweep signatures, its computational requirements are considerably reduced due to a novel, to our knowledge, approach that relies on SNP vectors to detect SFS and LD changes. A SNP vector is an entire alignment column, which the *μ statistic* employs as a unit, contrary to other neutrality tests that rely on summarized SNP-vector representations. To compute the *μ statistic*, we employ a SNP-driven, sliding-window algorithm that reuses calculated data between overlapping windows, thus considerably reducing the execution time. Consecutive SNP windows with variable size in terms of bps are placed along a dataset to allow exhaustive scans without requiring user-defined parameters. This achieves increased granularity in SNP-dense regions and avoids redundant operations in SNP-sparse ones. Consequently, processing speed improves without deteriorating the quality of the results. We release RAiSD (Raised Accuracy in Sweep Detection), an open-source software tool that combines the computationally inexpensive sliding-window algorithm for the *μ statistic* with a memory-aware approach for parsing input data. RAiSD allocates a negligible amount of memory (typically few MBs) irrespectively of the dataset size, thus maintaining overall low memory requirements. Here, we thoroughly scrutinize RAiSD performance on detecting and localizing selective sweeps in non-equilibrium evolutionary models, presenting results for bottlenecked populations, migration models, recombination models, background selection, and soft sweeps.

## Results

### Experimental setup

To evaluate RAiSD, we conducted a series of test runs employing a total of 113 simulated datasets, with the aim to assess its power to accurately localize hard selective sweeps, and its robustness to confounding factors such as population bottlenecks, migration, recombination heterogeneity, and background selection. Table 1 provides a succinct description of the datasets. Each dataset comprises 1000 neutral sets of SNPs, and 1000 sets of SNPs with a sweep and/or a confounding factor, while the sample size is 20 sequences. We assessed performance in terms of (i) accuracy (reported distance from the true selection target), (ii) sensitivity, i.e., the true positive rate (TPR), (iii) false positive rate (FPR), (iv) success rate of the detection process, i.e., the proportion of sweeps detected within a given maximum distance from the sweep location, and (v) execution time. We compared RAiSD with state-of-the-art software tools that detect selective sweeps, such as SweepFinder2^8^, SweeD^9^, and OmegaPlus^10^. Note that RAiSD evaluates locations based on a SNP-driven, sliding-window algorithm, whereas SweeD, OmegaPlus, and SweepFinder2 rely on a grid of equidistant locations of user-defined size. Therefore, to facilitate comparisons, we interpolated the scores of RAiSD based on the grid locations of the other tools.

**Table 1:** Summarized description of the simulated datasets used for experimental evaluation (see Supplementary Data 1 for command lines)

Dataset #	Sweep type	Confounding factor	Simulation software	Varying parameters	Range of values
	Hard, complete	Bottleneck	Hudson’s ms and mssel	Severity (*−eN*)	0.005–0.5
1–60				Duration (*−eN*)	80–400 (4*N*_0_ generations)
				Beginning (−*eN*)	800–20,000 (4*N*_0_ generations)
61–70	Hard, complete	Migration	Hudson’s ms and mssel	Population join (*−ej*)	0.003–3 (4*N*_0_ generations)
71–80	Soft, complete	None	Discoal	Drift until frequency (-*f*)	0.1–1.0
81–101	No sweep	Recombination	msHOT	Hotspot region size (*−v*) Hotspot intensity (*−v*)	5–10 kb 2–100
102–111	Hard, complete	Recombination	msHOT and mbs	Hotspot region size (*−v*) Hotspot intensity (*−v*)	5 kb 2–20
112–113	Hard, complete	Background selection	SFS_CODE	a) Set of SNPs with demography b) Set of SNPs with demography + background selection c) Set of SNPs with demography + background selection + sweep	

### Bottleneck models

Employing the software tools ms^12^ and mssel (kindly provided by R.R. Hudson), we simulated 60 bottlenecks (Table 1, datasets 1–60), each comprising both neutral sets of SNPs and sets of SNPs with demography and selection at the center of the simulated region. The bottleneck severity (the relative population size during a bottleneck compared to the present-day population size) ranged from 0.5 to 0.005, while the bottleneck duration varied between 80 and 400 generations, and the beginning of the bottleneck (backward in time) was between 800 and 20,000 generations. To convert coalescent time units to generations, we assumed that the present-day population size consists of 50,000 haploid genomes.

**Fig. 1:**
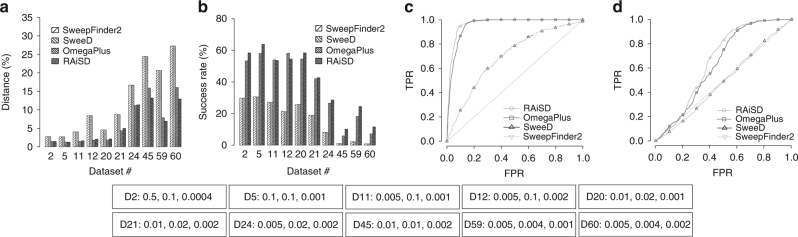
RAiSD evaluation and comparison with SweepFinder2, SweeD, and OmegaPlus, for a subset of the bottleneck models. **a** Detection accuracy, measured as the average distance between the reported locations and the known target of selection (reported as a percentage over the region length). **b** Success rate, reported as the percentage of the runs with best-score location in the proximity (closer than 1% of the region length) of the known target of selection. **c** ROC curve for bottleneck model 45. **d** ROC curve for bottleneck model 60. The parameters that vary per model are provided below the plots in the form: “D[#]: [severity], [begin time], [duration]”

Figure 1 shows that all tools perform well when analyzing datasets with mild bottlenecks. The average reported distance (Fig. 1a) between the true and the predicted selection targets for dataset 2, for instance, is as low as 2.5% for SweepFinder2 and SweeD, and 1.4% for OmegaPlus and RAiSD. Success rates (Fig. 1b) are also high, with SweepFinder2 and SweeD achieving 29.8%, while OmegaPlus and RAiSD deliver 53.5% and 59.0%, respectively. Overall, RAiSD and OmegaPlus exhibit comparable performance, outperforming SweepFinder2 and SweeD for all datasets, with RAiSD achieving higher accuracy than OmegaPlus in 40 out of the 60 different bottleneck models, and higher success rates in 57 out of the 60 execution cases^13^. More importantly, RAiSD achieves superior performance for the most severe bottlenecks, i.e., datasets 45, 59, and 60. For dataset 60, for instance, SweepFinder2 and SweeD exhibit totally random behavior in terms of accuracy: since the true sweep location is at the center of the chromosome, we expect neutrality tests that report uniformly distributed positions to detect sweeps at a distance of 25% of the total chromosome length far from the true selection target (on average). Both SweepFinder2 and SweeD report positions at a distance of 27.3%, while OmegaPlus and RAiSD report positions at distances of 16.0% and 12.9%, respectively. With respect to the success rate, only 0.8% of the predicted targets by SweepFinder2 and SweeD are within 10 kb (1% of the 1-Mb region size) from the true selection target. OmegaPlus reports that 7.2% of the targets are within the 10-kb range, whereas RAiSD reports 11.6%.

Figures 1c and 1d illustrate ROC curves for datasets 45 and 60, respectively. While mild bottlenecks pose little challenge to all the tools, with TPR values (FPR = 5%) for dataset 2, for instance, as high as 97.8% for SweepFinder2 and SweeD, and 99.8% for OmegaPlus and RAiSD, sensitivity drops dramatically as the bottleneck severity and duration increase (datasets 45 and 60). RAiSD steadily exhibits higher sensitivity than SweepFinder2 and SweeD, while yielding similar performance to OmegaPlus. The respective TPR values (FPR = 5%) for dataset 60 are as low as 4.1% for SweepFinder2 and SweeD, 5.1% for OmegaPlus, and 5.8% for RAiSD, which are indicative of the challenges that severe bottlenecks pose to sweep detection. It should be noted that in some of the cases of old and severe bottlenecks (datasets 55–58), OmegaPlus outperformed RAiSD, presumably due to the fact that the former examines all genomic regions surrounding a given location to find the configuration that maximizes the score of the test.

### Migration models

For the models with migration (Table 1, datasets 61–70), we implemented a continent-island model where the effective population size of the island is 20 times smaller than the continent effective population size. The continent acts as a ghost population, therefore only sequences from the island are available. The migration rate *M*_ic_ was set to 3 ($M_{{\mathrm{ic}}} = 4N_{\mathrm{c}}m_{{\mathrm{ic}}}$, where *N*_c_ is the effective population size of the continent and *m*_ic_ is the proportion of the island population that consists of immigrants from the continent). The continent and the island merged into a common population at time *t*_merge_, measured in the usual coalescent timescale, i.e., in 4*N*_c_ generations. In simulations, the time *t*_merge_ ranged from 0.003 to 3, implementing population divergence models that ranged from very recent (0.003) to very old (3). Both the population mutation rate *θ* and the recombination rate *ρ* were 2000 for the whole genomic region.

**Fig. 2:**
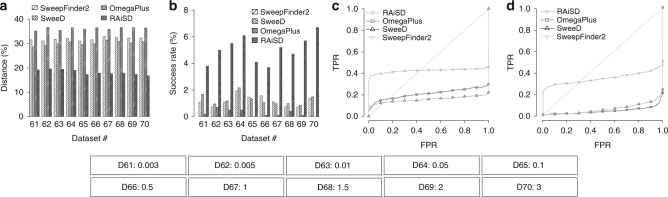
RAiSD evaluation and comparison with SweepFinder2, SweeD, and OmegaPlus, for the migration models. **a** Detection accuracy, measured as the average distance between the reported locations and the known target of selection (reported as a percentage over the region length). **b** Success rate, reported as the percentage of the runs with best-score location in the proximity (closer than 1% of the region length) of the known target of selection. **c** ROC curve for migration model 61. **d** ROC curve for migration model 70. The parameter that varies per model is provided below the plots in the form: “D[#]: [population join time]”

Figure 2 shows that the SFS-based methods (SweepFinder2 and SweeD) outperform the LD-based one (OmegaPlus), while RAiSD achieves the highest performance in terms of accuracy (Fig. 2a) and success rate (Fig. 2b). In fact, the behavior of each tool is similar irrespectively of the migration parameters. SweepFinder2, SweeD, and OmegaPlus report sweep locations at average distances of 31.9%, 30.1%, and 36.1%, respectively, whereas RAiSD’s accuracy is 18.1%, yielding 2 and 1.66 times more accurate scans than OmegaPlus and SweeD/SweepFinder2, respectively. With respect to the success rate, RAiSD reports sweep locations in the proximity of the true selection target in 5.05% of the cases, whereas the respective rates for SweepFinder2, SweeD, and OmegaPlus are 1.18%, 1.27%, and 0.26%.

Figures 2c and 2d illustrate ROC curves for datasets 61 and 70, respectively (the population divergence times are 0.003 for dataset 61 and 3 for dataset 70). The area under RAiSD’s ROC curve is clearly larger than the respective areas under the ROC curves of SweepFinder2, SweeD, and OmegaPlus, demonstrating RAiSD’s higher sensitivity. For low FPR values, such as 1% for instance, RAiSD’s sensitivity is as high as 36.7%, whereas SweepFinder2, SweeD, and OmegaPlus achieve 8.6%, 8.7%, and 6.6%, respectively. Overall, the ROC curves demonstrate a plateau behavior, which suggests that TPR values do not increase any further once a particular FPR value is reached. Such behavior shows that, as FPR increases, a large number of runs yield higher scores for neutral datasets than for datasets with selection. This observation suggests that (a) the tests used in this study do not detect some of the signatures generated by the combined effect of migration and selection, and (b) migration can generate signatures that resemble those of a selective sweep. In general, migration models pose severe challenges to existing sweep detection methods, suggesting that appropriate sweep signatures for migration models are yet to be found.

### Recombination hotspots

Due to the fact that recombination heterogeneity affects the extent of LD^14^, we conducted a series of experiments with recombination hotspots to examine the effect of recombination heterogeneity on the scores calculated by RAiSD, SweeD, SweepFinder2, and OmegaPlus. We initially assessed the effect of recombination heterogeneity on FPR by employing the software tool msHOT^15^ (Table 1, datasets 81–101) to simulate recombination hotspot models with recombination intensity that ranged from 2 to 100 (relative to the rest of the genome) and hotspot region sizes of 5 kb and 10 kb, as well as a model with three hotspot regions of 5 kb each. We set cutoff values (at the 95th percentile) based on neutral models without recombination hotspots, and examined FPR values using the neutral models with recombination hotspots. Table 2 reveals that the effect of recombination hotspots on FPR is minimal. In fact, the average FPR values for SweepFinder2, SweeD, OmegaPlus, and RAiSD are 3.76%, 3.72%, 5.65%, and 5.01%, respectively.

**Table 2:** The effect of recombination hotspots on FPR

Hotspot region size	5 kb							10 kb							5 kb × 3						
**Recombination intensity**	**2**	**4**	**8**	**10**	**20**	**40**	**100**	**2**	**4**	**8**	**10**	**20**	**40**	**100**	**2**	**4**	**8**	**10**	**20**	**40**	**100**
SweepFinder2	3.9	3.1	4.3	4.4	4.9	4.0	3.8	3.8	4.1	3.1	3.6	2.6	4.1	4.2	3.7	4.8	3.2	3.6	3.5	3.0	3.3
SweeD	3.9	3.1	4.2	4.4	4.9	3.9	3.7	3.7	4.1	3.1	3.5	2.6	4.1	4.2	3.7	4.8	3.1	3.6	3.5	2.9	3.3
OmegaPlus	5.9	5.2	4.3	5.4	6.8	5.0	7.0	5.1	6.6	6.0	6.2	5.4	4.6	5.9	5.6	5.7	5.8	5.4	5.7	5.0	6.1
RAiSD	5.1	5.5	5.4	5.5	6.3	4.6	5.6	6.4	4.8	5.7	4.0	4.6	4.9	5.2	4.9	4.5	4.9	3.1	5.5	4.1	4.6

We used three configurations of hotspot region size and seven different recombination intensity values. The results suggest that the FPR is relatively unaffected for all tools (cutoff values set at the 95th percentile)

**Fig. 3:**
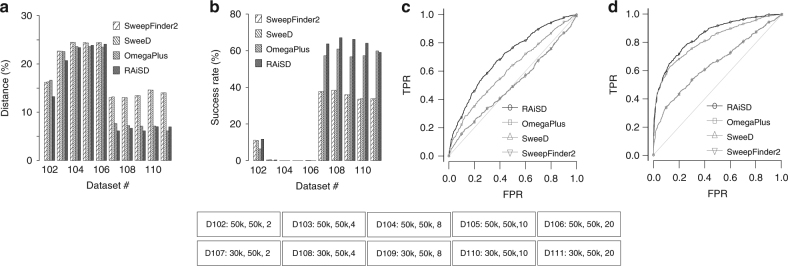
RAiSD evaluation and comparison with SweepFinder2, SweeD, and OmegaPlus, for the models with a recombination hotspot and a selective sweep. **a** Detection accuracy, measured as the average distance between the reported locations and the known target of selection (reported as a percentage over the region length). **b** Success rate, reported as the percentage of the runs with best-score location in the proximity (closer than 1% of the region length) of the known target of selection. **c** ROC curve for recombination model 102 (sweep location: 50 kb, center of recombination hotspot: 50 kb). **d** ROC curve for recombination model 107 (sweep location: 30 kb, center of recombination hotspot: 50 kb). The parameters that vary per model are provided below the plots in the form: “D[#]: [sweep location], [center of hotspot], [recombination intensity]”

Furthermore, we assessed the effect of recombination hotspots on TPR by additionally employing the software tool mbs^16^ to simulate recombination hotspot models with a selective sweep (Table 1, datasets 102–111). We set cutoff values (at the 95th percentile) based on neutral models with a recombination hotspot, and examined TPR using recombination hotspot models with a selective sweep. We employed a series of models with a 5-kb recombination hotspot region and increasing recombination intensity that ranged from 2 to 20. We did not consider additional values for recombination intensity due to the prohibitively long run times of mbs^16^. Figure 3 shows the performance of RAiSD and the rest of the tools when a selective sweep is simulated at the center of the hotspot region (datasets 102–106), as well as when it occurs at some distance outside the hotspot region (datasets 107–111). In the case of a sweep occurring inside the hotspot region, all tools exhibit gradually worsening performance as recombination intensity increases, with the average distance from the true target of selection reaching around 25% for all tools (Fig. 3a, datasets 102–106), and the success rates in terms of detected sweeps at a distance closer than 1 kb (1% of the simulated region size) from the true selection target becoming lower than 1% (Fig. 3b, datasets 102–106). When a sweep occurs outside the hotspot region, all tools exhibit considerably higher detection capacity, with RAiSD and OmegaPlus outperforming SweepFinder2 and SweeD, both in terms of average distance (Fig. 3a, datasets 107–111) and success rate (Fig. 3b, datasets 107–111). While RAiSD outperforms OmegaPlus in most of the cases, it should be noted that in the case of the highest recombination intensity considered here (dataset 111), RAiSD achieves an average distance of 6.9% and a success rate of 59.10%, whereas OmegaPlus achieves 6.1% and 59.89%. Figures 3c and 3d illustrate ROC curves for datasets 102 and 107, respectively, revealing that all tools achieve higher TPR when a sweep is not occurring inside the hotspot region. For dataset 102 and FPR 5%, RAiSD, OmegaPlus, SweeD, and SweepFinder2 achieve TPR of 18.6%, 13.5%, 9.0%, and 9.0%, respectively, whereas for dataset 107 the respective TPR are 48.6%, 47.5%, 23.3%, and 23.2%.

### Background selection

The hitchhiking theory^4^ predicts that a recent selective sweep reduces the amount of genetic diversity in regions with reduced recombination rate^17–19^. A similar prediction holds for background selection^20^. To assess the performance of RAiSD and the other tools/tests in the presence of background selection, we performed the following experiments. Initially, we employed a dataset (Table 1, dataset 112) that comprised 1000 neutral sets of SNPs without background selection (to calculate the top 5% cutoff value per test) and 1000 neutral sets of SNPs with background selection. We could therefore evaluate the FPR, since no selective sweeps were present and all results that exceeded the cutoff value were false positives. Thereafter, we assessed sensitivity (TPR) by employing a dataset (Table 1, dataset 113) that comprised 1000 neutral sets of SNPs with background selection (to calculate the top 5% cutoff value per test) and 1000 sets of SNPs with both background selection and a selective sweep. We used the software SFS_CODE^21^ to simulate 100-kb genomic regions similarly to Maher et al.^22^. Maher et al. studied the population genetics of rare variants of genes associated with complex diseases using the results of Torgerson et al.^23^ to assign a probability of negative selection in coding and non-coding regions of each gene, the human demographic model of Tennessen et al.^24^, and a distribution of selection coefficients that operate on non-synonymous mutations^25^.

Table 3 reveals that RAiSD outperforms the rest of the tools in terms of TPR (97.5%), accuracy (9.94%), and success rate (6.2% of the reported locations are within 1 kb from the true selection target), with the respective values for SweepFinder2, SweeD, and OmegaPlus being considerably lower. RAiSD, however, exhibits an FPR value that is as high as 37.1%, whereas the respective values for OmegaPlus, SweeD, and SweepFinder2 are 8.4%, 0.3%, and 0.3%. This indicates that, when background selection is not considered for the calculation of the cutoff value, RAiSD is sensitive to its effect, mainly due to the decrease of genetic variation. This does not affect the tool’s capacity to detect a selective sweep when there is one, as long as background selection is considered in the calculation of the cutoff value. The elevated FPR indicates that RAiSD is more sensitive to background selection than the other tools when not controlling for it, which is expected, since one of the *μ statistic* factors that contribute to RAiSD’s high accuracy and TPR is the *μ*^VAR^. This factor explicitly accounts for reduced genetic diversity, which, except for being a signature of a selective sweep, is also expected in the presence of background selection, and thus it is required to control for it in order to correctly detect selective sweeps. Using simulations with background selection but no selective sweeps to specify the cutoff value, one can control the FPR to the desired level (here 5%), in which case the proportion of the true selective sweeps that RAiSD detects is the highest among the rest of the tools.

**Table 3:** Performance evaluation of SweepFinder2, SweeD, OmegaPlus, and RAiSD in the presence of background selection.

	SweepFinder2	SweeD	OmegaPlus	RAiSD
FPR	**0.3**	**0.3**	8.4	37.1
TPR	59.9	56.5	21.1	**97.5**
Distance to sweep	24.47	25.33	26.89	**9.94**
Success rate	2.0	1.9	2.3	**6.2**

Values in boldface indicate the best performance per metric

### The effect of model misspecification

**Fig. 4:**
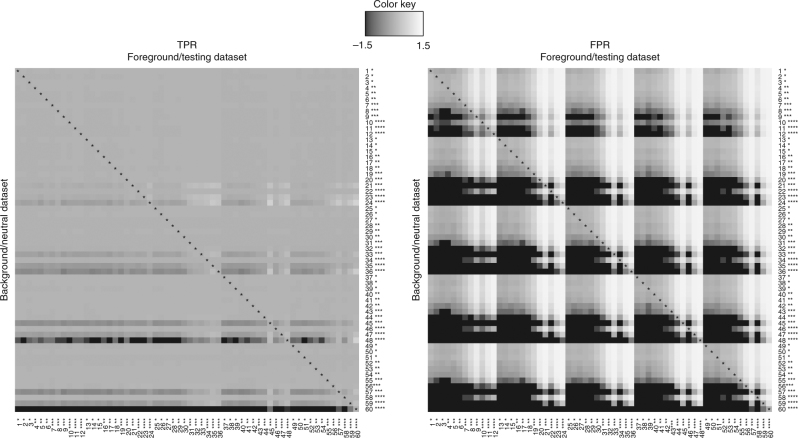
Evaluation of RAiSD performance in terms of TPR (left panel) and FPR (right panel) when the background/neutral (or base) model for the 60 bottleneck models is misspecified. The number of ‘*’ characters next to the dataset number represents the reduction of the population size during the bottleneck phase. The colors of the tiles in the TPR heatmap represent the ${\rm log}_{10}(P_{{\rm TT}}/P_{{\rm TA}})$, where *P*_TT_ is the TPR when the foreground and background models match (diagonal), and *P*_TA_ is the measured TPR for the foreground model. In the FPR heatmap, the colors represent the ${\rm log}_{10}(P_{{\rm FT}}/P_{{\rm FA}})$, where *P*_FT_ is the FPR when the foreground and background models match, and *P*_F*A*_ is the measured FPR for the foreground model. Thus, darker/lighter tiles than the one on the diagonal indicate the effect of model misspecification toward smaller/larger rates than the one calculated when the demographic model is correctly inferred. The TPR heatmap reveals that, when a bottleneck is assumed as the null model, the TPR is not greatly affected, even if a bottleneck is not present in the evaluation dataset. The FPR heatmap, however, suggests that bottlenecks generate a high number of false positives when not taken into account

The typical evaluation approach in the current study relies on a cutoff value per neutrality test, calculated based on datasets that incorporate all the evolutionary forces except for a selective sweep. Thus far, we evaluated the performance of all tools assuming that the demographic model was correctly estimated. In addition, we assessed the effect of model misspecification on the detection process by examining how the FPR and TPR values are affected when the model used for the calculation of the cutoff value differs from the one used for the detection of the selective sweep. For this purpose, we employed the 60 datasets with bottlenecks (Table 1, datasets 1–60), and for each neutrality test, we used the cutoff values that were calculated based on the neutral sets of SNPs (per dataset) to evaluate both the FPR and the TPR for all 60 datasets. Figure 4 illustrates the results for RAiSD in a 60 × 60 heatmap (see Supplementary Fig. 1 for SweepFinder2, SweeD, and OmegaPlus), where the diagonal corresponds to the “ideal” TPR or FPR calculations, i.e., when the demographic model is correctly inferred. Off-diagonal elements represent the effect of model misspecification in relation to the corresponding diagonal value. The value in cell (*i*, *j*) in the FPR heatmap, for instance, is the $log_{{\mathrm{10}}}({\mathrm{FPR}}_{ij}/{\mathrm{FPR}}_{ii})$, where FPR_*ij*_ is the FPR when model *i* is used for the calculation of the cutoff value and model *j* is used for the evaluation of the selective sweep. The number of ‘*’ characters accompanying the dataset number represents the population size reduction during the bottleneck phase.

One can observe that when datasets without bottlenecks are used to calculate the cutoff value and a bottleneck has occurred in the evaluation datasets, the FPR increases dramatically (even 10 times higher than the 5% expected value; light-colored columns in the FPR heatmap). Inversely, the FPR decreases dramatically (below the 5% expected value) when a (severe) bottleneck model is used for the cutoff value calculation and no (severe) bottleneck has occurred in the evaluation dataset (black regions in the FPR heatmap). On the contrary, the TPR is relatively robust to model misspecification for the majority of the bottleneck models, apart from the most severe ones. This is due to the fact that true selective sweep regions are mostly characterized by very high RAiSD scores, which typically exceed the top 5% cutoff value. Severe bottlenecks (e.g., datasets 48 and 60), however, can also produce notably high scores, even in the absence of a selective sweep. We find that it is beneficial to assume the occurrence of a bottleneck in the calculation of the cutoff value, since that way the FPR is controlled near the 5% expected value whereas the TPR remains largely unaffected.

### Soft selective sweeps

**Fig. 5:**
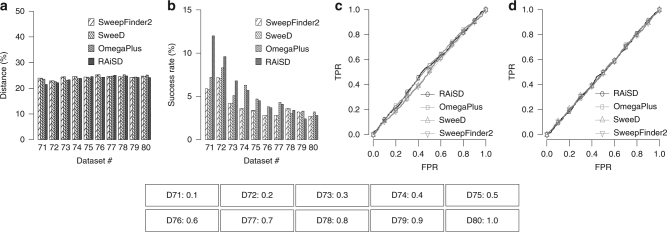
Soft sweeps cannot be detected with hard-sweep detection methods. Even if a mutation starts to be beneficial at frequency 0.1, hard selective sweep methods behave as random classifiers and are unable to accurately detect the sweep location. **a** Detection accuracy, measured as the average distance between the reported locations and the known target of selection (reported as a percentage over the region length). **b** Success rate, reported as the percentage of the runs with best-score location in the proximity (closer than 1% of the region length) of the known target of selection. **c** ROC curve for dataset 71. **d** ROC curve for dataset 75. The parameter that varies per model is provided below the plots in the form: “D[#]: [frequency at which mutation starts being beneficial]”

RAiSD is designed to detect hard selective sweeps. To test how RAiSD and the other tools perform for soft selective sweeps, we generated datasets using the software tool Discoal^26^. We simulated 100-kb genomic regions with a soft selective sweep at the center. The time of fixation (backwards in time) was set to 0.001 and the selection intensity *a* was set to 20 (*α* = 2*N*s, where *N* is the effective population size and *s* is the selection coefficient). A total of 10 datasets (Table 1, datasets 71–80) were generated, with the initial frequency at which a previously neutral allele became beneficial varying from 0.1 to 1.0. As illustrated in Fig. 5, all tools perform poorly in terms of accuracy, success rate, and TPR. All tools report around 25% average distance from the true selection target, which corresponds to randomly selected locations since the center of the sweep is at the center of the simulated region. The success rates, in terms of detected sweeps at a distance closer than 1 kb (1% of the simulated region size), are similarly low (< 12%) for all the tests, whereas the ROC curves resemble the behavior of a random classifier.

### Complex sweep-detection workflows

Recent studies propose complex workflows that combine multiple signatures of a selective sweep. The Composite of Multiple Signals^27^ (CMS) tool identifies three distinct signatures of selection: long-range haplotypes, differentiated alleles, and high-frequency derived alleles. Thus, it does not only employ classical selective sweep signals, but also signatures of local adaptation (differentiated alleles and long-range haplotypes). S/HIC^28^ is a supervised learning software that first “learns” to recognize patterns of variation from simulations and then it can be used to classify an unknown genomic dataset into one of the following classes: (i) neutral, (ii) linked soft, (iii) soft sweep, (iv) linked hard, or (v) hard selective sweep. Linked soft and linked hard regions are neutral regions in the proximity (thus linked) of a soft and a hard selective sweep, respectively. The power of S/HIC lies in the fact that a mathematical formulation of the likelihood of a sweep (as employed in SweeD and SweepFinder/SweepFinder2) is not required, since the classifier relies on simulations to “learn” the properties of each model. This approach, however, is sensitive to the training process, particularly with respect to model misspecification.

We compared RAiSD (and the other tools) with S/HIC and CMS, testing S/HIC using simulated and real datasets, and CMS using real datasets. A major disadvantage of both CMS and S/HIC is that they contain complex pipelines that can yield their application cumbersome. For instance, S/HIC ideally requires separate training for each demographic model. Since a thorough analysis of S/HIC is beyond the scope of the current study, we employed the training set provided by the developers of S/HIC^28^, which is relevant to the European demography, to test against all 60 bottleneck datasets in our study. Hence, the results of S/HIC on the simulated datasets were affected by model misspecification. Due to the fact that S/HIC classifies subgenomic regions as either hard sweep, soft sweep, linked hard, linked soft, or neutral, we superimposed the results of RAiSD and the other tools on the genome map annotated by the results of S/HIC. We observed that S/HIC classified several regions in the 60 simulated datasets as either soft selective sweeps or linked soft, even though no soft selective sweeps were present (Supplementary Figs. 2 and 3). For comparisons based on real data, we used the results for the YRI population of the 1000 Genomes dataset that are provided by the S/HIC developers (https://github.com/kern-lab/shIC), as well as the respective ones provided by the CMS^27^ authors. Due to the fact that CMS reports scores at certain locations along the chromosome, we interpolated the results of RAiSD using the positions of CMS to facilitate comparisons. We found that the results obtained by RAiSD^29^ (as well as the other tools tested in this study) differ considerably from those obtained by S/HIC and CMS.

### 1000 Genomes dataset

**Fig. 6:**
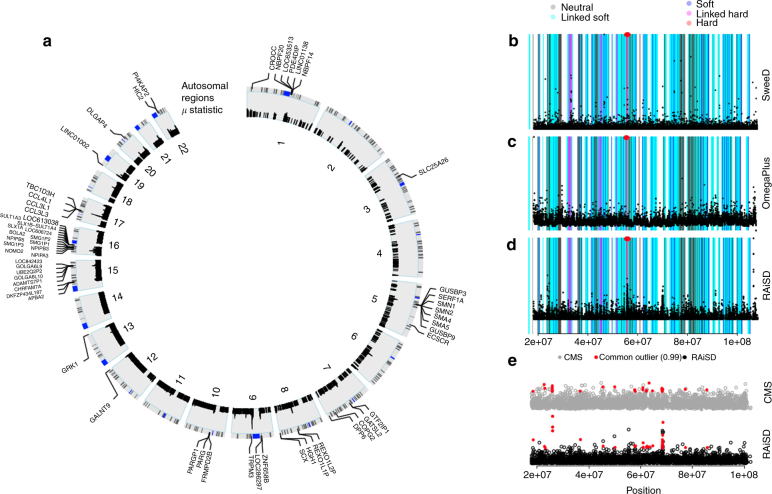
Detection of selective sweeps in the YRI population. **a** Selected top genes (threshold set to 99.95%) identified as targets of positive selection for all chromosomes in the YRI population (full list provided in Supplementary Table 1). **b-d** The results of SweeD, OmegaPlus, and RAiSD for chromosome 14, in comparison with the results of S/HIC. S/HIC is a machine learning tool, thus each region is classified as neutral (dark-gray), linked soft (light blue), soft (purple), linked hard (pink), and hard (red). For clarity, hard-sweep regions are also denoted by a red dot at the top of each plot. **e** Common outliers between CMS and RAiSD for chromosome 14

We analyzed the 1000 Genomes dataset^11^ using RAiSD to demonstrate the tool’s capacity to handle real data. It is currently one of the largest publicly available datasets, both in terms of sample size and number of SNPs, comprising 2504 human samples from 26 populations and a total of 77,832,252 SNPs for the whole set of autosomes (phase 3). We employed RAiSD to scan each chromosome of the YRI population separately, excluding chromosomes X and Y. We also compared the results of RAiSD^29^ with the results of OmegaPlus, SweeD, and SweepFinder2, as well as S/HIC and CMS. Figure 6a illustrates RAiSD scores per autosome of the YRI population. For the sake of clarity, the figure shows only the points with the highest *μ statistic* values (>99.5%). Centromere regions (coordinates obtained from UCSC^30^) and their flanking regions, corresponding to 5% of each chromosomal length, were excluded from the analysis. We provide a short list of the 60 genes with the highest scores (top 0.05%) from all chromosomes in Supplementary Table 1. Figure 6b–d illustrates the results of SweeD, OmegaPlus, and RAiSD for the analysis of chromosome 14, superimposed on the annotated chromosomal map of S/HIC, whereas Fig. 6e provides a comparison between RAiSD and CMS for the same chromosome.

## Discussion

To the present day, most neutrality-test evaluations solely examine robustness to bottlenecks. In this study, we showed that migration also poses major challenges to selective sweep detection, and that neutrality tests are not robust when applied on datasets from structured populations. Since it is unrealistic to assume complete isolation of populations for most species, the effect of migration on sweep detection needs to be further examined. Knowing the population structure (and generally the demographic model) allows to set a significance threshold, thus helping to control the FPR.

Besides demography, real data pose additional challenges to selective sweep detection. The motivation for a sliding window with a fixed number of SNPs derives from the assumption that the population mutation rate *θ* and the population recombination rate *ρ* remain approximately constant along a chromosome. Consequently, the fixed-size window would correspond to a narrower distribution of the number of recombination events as compared to an unconditional one, and the emerging SNP patterns would mostly be a function of the genealogy. In real datasets, however, the assumption may be violated, since some genomic regions may be recombination hotspots or exhibit higher mutation rates than others. Furthermore, low-complexity regions and the centromere make sequencing difficult and should ideally be excluded from the analysis. To facilitate real-data analyses, RAiSD reports the values of all three factors that form the *μ statistic*. It was empirically determined that the product of these factors gives superior results. One, however, may opt to employ a combination of these factors and/or to assign a different weight to each one of them. For instance, to give a lower weight to *μ*^VAR^ in the case that *θ* varies extensively along the chromosome, one may calculate the *μ statistic* as $(\mu ^{{\mathrm{VAR}}})^k \times \mu ^{{\mathrm{SFS}}} \times \mu ^{{\mathrm{LD}}}$, where 0 ≤ *k* < 1.

The same approach can be followed in the case of background selection, which reduces the levels of genetic diversity locally in the genome similarly to a selective sweep^31–33^. Background selection has been extensively studied in humans^34^ and Drosophila^35^. It has been previously asserted that if one considers adaptation as a fine tuning of genotypes to their environment, most mutations with detectable fitness effects observed in a well-adapted population will be deleterious^36^. RAiSD controls the effect of background selection via a threshold-adjustment process that relies on simulations with background selection but no selective sweeps. The tool’s susceptibility to background selection, when neglected in the model, is due to the local effect of background selection on the reduction of polymorphisms, which affects the *μ*^VAR^ factor.

When real data were analyzed, i.e., the YRI population, RAiSD showed considerably different behavior than the complex detection workflow of S/HIC, with high RAiSD values mostly not corresponding to S/HIC-characterized hard-sweep regions (Supplementary Fig. 4). In fact, S/HIC results were also largely different than the rest of the tools, with S/HIC identifying most of the chromosomal regions as either soft sweeps or linked-soft. It should be noted, however, that RAiSD, S/HIC, and SweeD detected one genomic region in chromosome 14 as a hard sweep. When S/HIC was used on simulated datasets without soft sweeps (datasets 1–60), using the European demographic model of Tennessen et al.^24^ in the training datasets, at least one linked-soft or soft-sweep region was identified in more than 25% of the datasets (Supplementary Figs. 2 and 3). In addition, S/HIC was greatly affected by demography since the vast majority of neutral datasets with strong bottlenecks were characterized as having some sort of selection (linked soft, linked hard, soft, or hard), whereas a hard selective sweep was only detected in a small fraction of datasets with a hard selective sweep and a mild bottleneck. This suggests that proper training, particularly with respect to the demographic model, is a prerequisite for the successful deployment of machine learning methods.

Scanning the YRI population resulted in a list of candidate genes. Even though describing the properties and attributes of such genes may lead to the story-telling fallacy^37^, we report for some of them (among the top 0.05% results over the entire genome) what has been discovered in the literature (Supplementary Discussion).

## Methods

### The *μ statistic* for sweep detection

The *μ statistic* serves as a measure of positive selection by assuming high values in regions where variation resembles the signatures that a sweep leaves in a genome. It is constructed by three factors that collectively account for the expected reduction of variation in the region of a sweep, the shift in the SFS toward low- and high-frequency derived variants, and the emergence of a localized LD pattern characterized by high LD on each side of a beneficial mutation and low LD between loci that are located on different sides of the beneficial allele (Supplementary Fig. 4). All three factors rely on occurrences of SNPs and SNP-vector patterns rather than actual computations on SNP data, achieving considerable performance and scalability gains over state-of-the-art methods and tools.

**Fig. 7:**
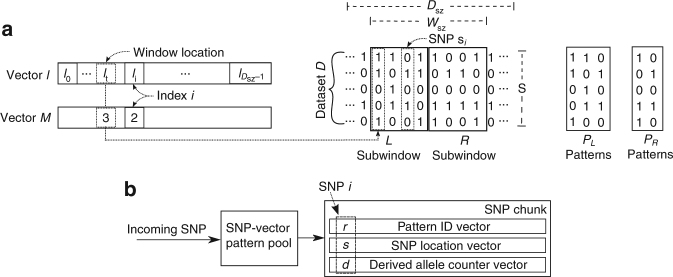
*μ statistic* computation example and the SNP-loading mechanism. **a** A window *W* of *W*_sz_ SNPs as considered for the calculation of the *μ statistic*. The location vector *l* and the derived-allele vector *M* correspond to the entire genomic region that is scanned, whereas the SNP window *W* is applied locally on *W*_sz_ SNPs following a sliding-window approach with a step of 1 SNP. **b** Schematic representation of the SNP-loading mechanism, as well as the SNP representation and the data structure on which RAiSD computes the *μ statistic*. Each SNP is matched in the SNP-vector pattern pool and a triplet of values (the pattern ID, the SNP location, and the number of derived alleles) is returned to the SNP-chunk data structure

Assume a dataset D with *S* individuals and *D*_SZ_ SNPs in a genomic region of length *D*_ln_ bp, as illustrated in Fig. 7a. Let *s*_*i*_ denote SNP with index *i* and *l*_*i*_ denote its location. To calculate the *μ statistic*, assume a fixed-size SNP window *W* with an even number of *W*_sz_ SNPs that is split into two non-overlapping subwindows of equal size, *L* and *R*. Let *P*_L_ and *P*_R_ be the two sets of SNP patterns found in the *L* and *R* subwindows, respectively. Also, let *M*(*s*_*i*_) be the number of individuals with derived alleles at SNP *s*_*i*_. The window *W* slides on the *D*_sz_ SNPs in *D* with step 1 SNP, calculating a total of $D_{{\mathrm{sz}}} - W_{{\mathrm{sz}}} + 1$ scores. Let index *t* of the first SNP in *W* also denote the window’s location on the *D*_sz_ SNPs. Therefore, for window *W* at position *t*, the *μ statistic* is computed as follows:

1 $$\mu _t = D_{{\mathrm{ln}}} \times \mu _t^{{\mathrm{VAR}}} \times \mu _t^{{\mathrm{SFS}}} \times \mu _t^{{\mathrm{LD}}},$$

based on the factors $\mu _t^{{\mathrm{VAR}}}$, $\mu _t^{{\mathrm{SFS}}}$, and $\mu _t^{{\mathrm{LD}}}$, which account for the three sweep signatures. The final *μ*_*t*_ score refers to genomic location $(l_{t + W_{{\mathrm{sz}}} - 1} + l_t)/2$, which corresponds to the middle of the distance (in bp) between the leftmost SNP in *W* (index *t*) and the rightmost one (index $t + W_{{\mathrm{sz}}} - 1$). The first factor, $\mu _t^{{\mathrm{VAR}}}$, quantifies the genetic variation in *W* and is computed as follows:

2 $$\mu _t^{{\mathrm{VAR}}} = \frac{{l_{t + W_{{\mathrm{sz}}} - 1} - l_t}}{{D_{{\mathrm{ln}}} \times W_{{\mathrm{sz}}}}},$$

where *l*_*t*_ and $l_{t + W_{{\mathrm{sz}}} - 1}$ are the locations (in bp) of the leftmost and rightmost SNPs in *W*, respectively. Thus, the numerator calculates the genomic length of *W* when the window is placed at location *t*. Given the fixed window size in terms of number of SNPs (*W*_sz_), the value of Eq. 2 is proportional to the window’s corresponding physical size, which means that the larger the region, the less the variation per bp for the fixed number of *W*_sz_ SNPs. The second factor, $\mu _t^{{\mathrm{SFS}}}$, captures the expected shift in the SFS as follows:

3 $$\mu _t^{{\mathrm{SFS}}} = \frac{{\mathop {\sum}\limits_{s_i \in W} [M(s_i) = 1] + \mathop {\sum}\limits_{s_i \in W} [M(s_i) = S - 1]}}{{W_{{\mathrm{sz}}}}},$$

where [] is the Iverson bracket notation, which returns 1 if the logical proposition expressed by the statement in brackets is true. Otherwise, it returns 0. Thus, Eq. 3 assumes high values with an increasing number of singletons (first sum) or SNPs with *S*–1 derived variants (second sum) in the window. Finally, the third factor, $\mu _t^{{\mathrm{LD}}}$, captures the third sweep signature via Eq. 4, computed as follows:

4 $$\mu _t^{{\mathrm{LD}}} = \frac{{\mathop {\sum}\limits_{s_i \in W_{\mathrm{L}}} [s_i \notin P_{\mathrm{R}}] \times \mathop {\sum}\limits_{s_i \in P_{\mathrm{L}}} [s_i \notin P_{\mathrm{R}}] + \mathop {\sum}\limits_{s_i \in W_{\mathrm{R}}} [s_i \notin P_{\mathrm{L}}] \times \mathop {\sum}\limits_{s_i \in P_{\mathrm{R}}} [s_i \notin P_{\mathrm{L}}]}}{{P_{L_{{\mathrm{sz}}}} \times P_{R_{{\mathrm{sz}}}}}},$$

where $P_{L_{{\mathrm{sz}}}}$ and $P_{R_{{\mathrm{sz}}}}$ are the sizes of *P*_L_ and *P*_R_ in number of SNP patterns, respectively. Equation 4 exhibits similar behavior with the well-studied *ω statistic* that utilizes LD. This is achieved, however, with considerably reduced computational complexity, by relying on the rationale that a low number of SNP patterns is expected in a region of high total LD, and vice versa. The numerator assumes high values with an increasing number of SNP-vector patterns that exist exclusively in one of the *L* or *R* subwindows, accounting for the expected low LD levels across a positively selected locus. For the example dataset in Fig. 7a, for instance, we have $\mathop {\sum}\nolimits_{s_i \in P_{\mathrm{L}}} [s_i \notin P_{\mathrm{R}}] = 2$ (number of SNP patterns in *P*_L_ that are not present in *P*_R_), and $\mathop {\sum}\nolimits_{s_i \in P_{\mathrm{R}}} [s_i \notin P_{\mathrm{L}}] = 1$ (number of SNP patterns in *P*_R_ that are not present in *P*_L_). Also, we have $\mathop {\sum}\nolimits_{s_i \in W_{\mathrm{L}}} [s_i \notin P_{\mathrm{R}}] = 3$ (number of SNPs in *W*_L_ that are not present in *P*_*R*_), and $\mathop {\sum}\nolimits_{s_i \in W_{\mathrm{R}}} [s_i \notin P_{\mathrm{L}}] = 2$ (number of SNPs in *W*_*R*_ that are not present in *P*_L_). The denominator assumes low values with a reduced number of SNP-vector patterns in each of the *L* or *R* subwindows, accounting for the expected high LD levels on each side of a positively selected site, thus increasing $\mu _t^{{\mathrm{LD}}}$. For the *P*_L_ and *P*_*R*_ sets of SNP patterns in Fig. 7a, we have $P_{L_{{\mathrm{sz}}}} = 3$ and $P_{R_{{\mathrm{sz}}}} = 2$.

The idea behind the fixed-size SNP window is that the distribution of the number of recombination events is narrow, conditioning on the number of SNPs. In a selective sweep region, the window size in bp would critically affect the results. On one hand, when selection is weak, multiple recombination events may have occurred in a given bp-based window and the sweep signal may have faded out. On the other hand, when selection is strong, it is probable that no SNPs are present and a window of variable size in bp is required (similarly to the window implementations in OmegaPlus, SweeD, and SweepFinder/SweepFinder2). This approach, however, becomes computationally expensive when large datasets are scanned. Adopting the assumption that the population mutation rate *θ* and the population recombination rate *ρ* remain approximately constant along a chromosome, and fixing the window size in terms of number of SNPs (since the number of SNPs is a visible measurement of the region), we aim to examine regions that do not vary wildly in terms of recombination events. Such regions will give more homogeneous signals for selective sweeps, facilitating their detection.

### The algorithm

RAiSD implements an iterative algorithm that conducts a varying number of sliding-window calculations per iteration. Each iteration entails a two-step process of fetching a number of SNPs from a secondary storage device, such as a hard drive, followed by a series of *μ statistic* computations per window location on the loaded set of SNPs. Figure 7b depicts the SNP-loading mechanism, as well as the SNP representation and the data structure on which the *μ statistic* calculations are conducted.

Every incoming SNP is initially compared with all existing SNP-vector patterns in a pattern pool. In the case that the entire pool is scanned and no matching pattern is found, the incoming SNP is stored in the pool as a new pattern. In every case, the pattern pool returns the respective SNP-vector pattern index (*r* in Fig. 7b) to the SNP-chunk data structure, along with the corresponding SNP location (*s* in Fig. 7b), and the number of derived alleles (*d* in Fig. 7b). The SNP-vector pattern pool is of fixed size in terms of memory requirements (1 MB), while the number of SNP-vector patterns it can accommodate depends on the SNP size, i.e., the number of samples. The SNP fetching step continues until the pattern pool is full, in which case the computational step for the *μ statistic* calculations begins.

As already mentioned, the *μ statistic* is calculated in a sliding-window manner, with multiple window iterations required to analyze the entire SNP chunk. The window step between iterations is 1 SNP, facilitating the sliding of the window on the three vectors that form the SNP chunk, as well as reducing the complexity for a data-reuse optimization. A pre-processing step initializes a set of base values for the three factors of the *μ statistic* based on the first window location for the chunk, with each subsequent window iteration simply updating these factors to account for the window shift. This achieves linear complexity throughout the SNP-chunk processing step and explains the considerable performance gains over existing implementations.

The two-step process of loading SNP data to the pattern pool and the SNP-chunk data structure, followed by the *μ statistic* calculations on the SNP chunk, is repeated as many times as required by the input dataset size. Prior to the re-initialization of the pattern pool with new SNP-vector patterns, a data relocation process is conducted in order to ensure that windows comprising SNPs that belong to neighboring SNP chunks are computed correctly. The relocation process is a prerequisite for correctness, due to the fact that only one pattern pool and one SNP-chunk data structure are kept in memory at all times, with each two-step iteration computing on the same memory space. This is one of the key factors that allow RAiSD to maintain negligible memory requirements irrespectively of the dataset size.

### Execution time

RAiSD and SweepFinder2, unlike SweeD and OmegaPlus, are sequential software tools, i.e., they do not have the capacity to employ multiple cores for faster execution. Furthermore, RAiSD execution times do not vary with input parameters, while this is not the case for the rest of the tools, yielding the outcome of execution time comparisons highly dependent on user-provided values for certain input parameters, such as “-s” for SweepFinder2, and “-grid” for SweeD and OmegaPlus. Such parameters enable faster runs by conducting less exhaustive scans (a sparser grid of evaluation points), potentially affecting the quality of the results. Hence, to provide an indicative performance comparison between RAiSD and the rest of the tools, we measured total execution times for the single-threaded analysis of a subset of 20 simulated datasets from the total of 113 datasets (Table 1). On average, over all runs, RAiSD yielded nearly 1000 times faster execution than SweepFinder2, 628 times faster execution than SweeD, and 32 times faster execution than OmegaPlus. The actual execution times and speedups, however, are of little significance since one can execute SweepFinder2, SweeD, and OmegaPlus arbitrarily faster or slower by reducing or increasing the grid size, respectively. It should be noted that, for all comparisons presented in this study, the grid-based tools were executed with a grid size of 1000 evaluation points.

### Scanning datasets with RAiSD

RAiSD is a readily available open-source implementation of the *μ statistic*. It combines a sliding-window processing algorithm with a memory efficient data parsing approach for ms^12^ and Variant Call Format (VCF) dataset formats in a stand-alone software tool implemented in C. The tool can be compiled using the provided *Makefile*. The basic input arguments for an analysis are a name for the run, and a path to the input file. RAiSD provides a set of parameters to facilitate downstream statistical analyses and calculate the FPR or TPR when simulated data are processed. By default, the software generates two output files. The first file contains dataset- and execution-related information, whereas the second file is a report that comprises the evaluated locations and the corresponding *μ statistic* scores. A set of optional parameters can be used to alter the default output mode, e.g., to generate multiple report files (one per dataset), or to suppress the inclusion of dataset-separator symbols in the report when a single input file contains multiple datasets, facilitating subsequent processing steps and downstream analyses (e.g., using R).

### Multi-tool analysis scripts and reproducibility

To facilitate sweep detection using multiple tools, such as the ones employed for evaluation purposes in this study, we implemented a series of bash scripts (available for download as part of the standard RAiSD release). These scripts were employed as-is for the experimental evaluations presented in this article and can therefore serve for reproducing the experiments. The provided scripts perform the following operations: (a) download and install the software tools RAiSD, OmegaPlus, SweeD, and SweepFinder2, (b) download and decompress the simulated datasets, (c) launch all software tools to analyze each dataset (and delete the input files upon completion), (d) parse the generated reports and create a per-dataset evaluation summary that comprises attained performance per tool, i.e., sensitivity, accuracy, success rate, and execution time, and (e) generate bar plots and ROC curves per tool per dataset.

### Code availability

The source code of RAiSD and a manual are available through GitHub at: https://github.com/alachins/raisd. The run scripts are available through GitHub at: https://github.com/alachins/raisd/tree/master/evaluation.

### Data availability

The simulated datasets are available at: 10.6084/m9.figshare.6324128 (datasets 1–70), and 10.6084/m9.figshare.6339095 (datasets 71–113). Comparison plots between RAiSD and the other tools based on datasets 1–60 are available at: 10.6084/m9.figshare.6340991. Comparison plots between RAiSD and the other tools based on the YRI population (1000 Genomes project) are available at: 10.6084/m9.figshare.6353045.

## Electronic supplementary material

Supplementary file

Description of additional supplementary items

Supplementary Data 1
