# Supplementary material for: RAiSD detects positive selection based on multiple signatures of a selective sweep and SNP vectors
Source: Commun Biol. 2018 Jun 27;1:79. doi: 10.1038/s42003-018-0085-8 (PMC6123745; doi:10.1038/s42003-018-0085-8)
Supplement: Supplementary file 1 — Supplementary file [file 42003_2018_85_MOESM1_ESM.pdf]

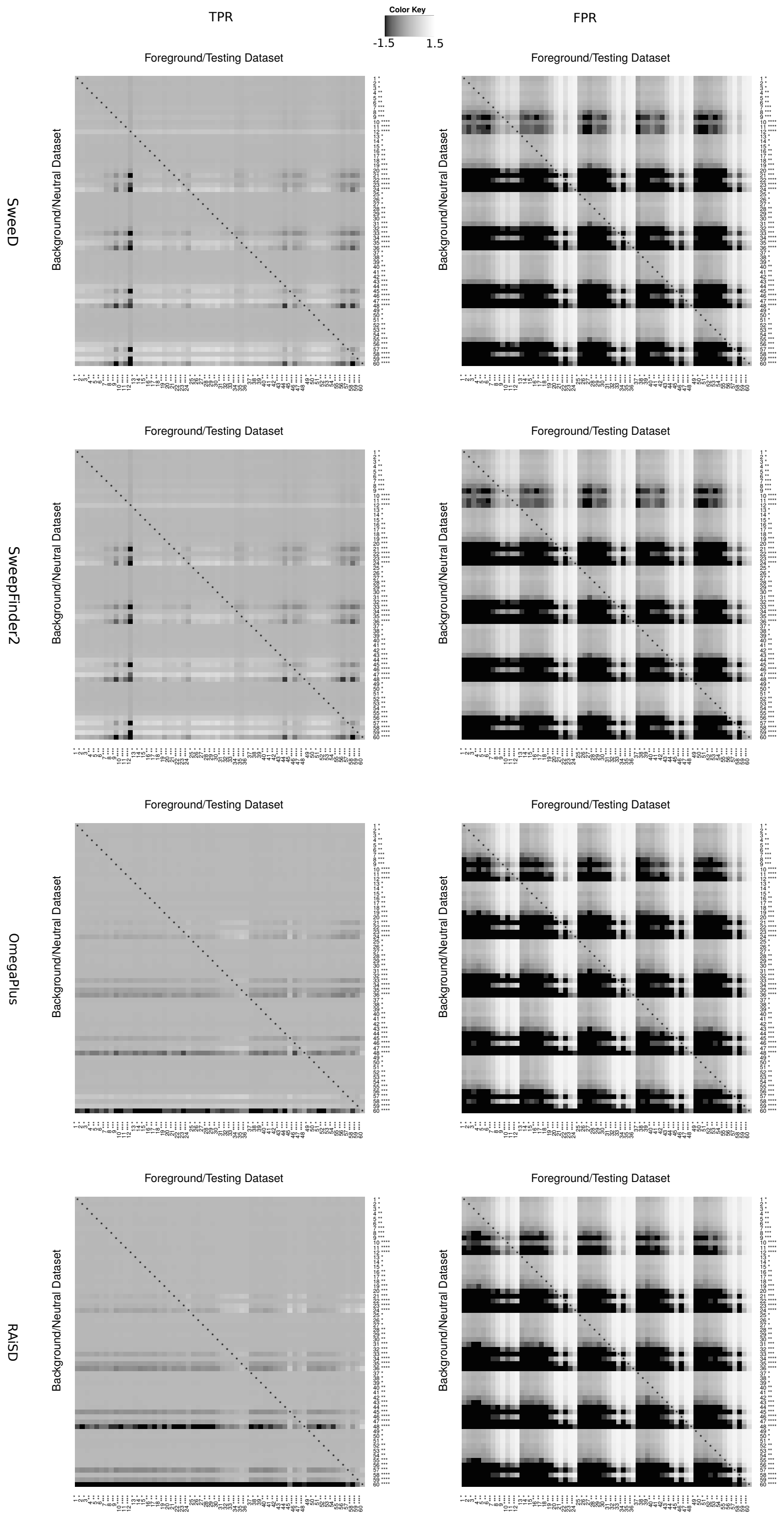

Supplementary Figure 1: **True positive rate (TPR) and false positive rate (FPR) for the 60 demographic models with bottlenecks (datasets 1–60) for SweeD, SweepFinder2, OmegaPlus, and RAiSD.** Neutral models (y-axis) were used to calculate the significance threshold. The TPR is calculated using models with selection (x-axis, TPR heatmaps), and the FPR is calculated using models without selection (x-axis, FPR heatmaps). The diagonal corresponds to the case where both the neutral and the selection (neutral for calculating the FPR) models come from the same demographic scenario. Off-diagonal elements have been scaled relatively to the diagonal. Darker gray tones represent cases where TPR/FPR is lower than the diagonal, whereas lighter gray tones represent cases where TPR/FPR is greater than the diagonal.

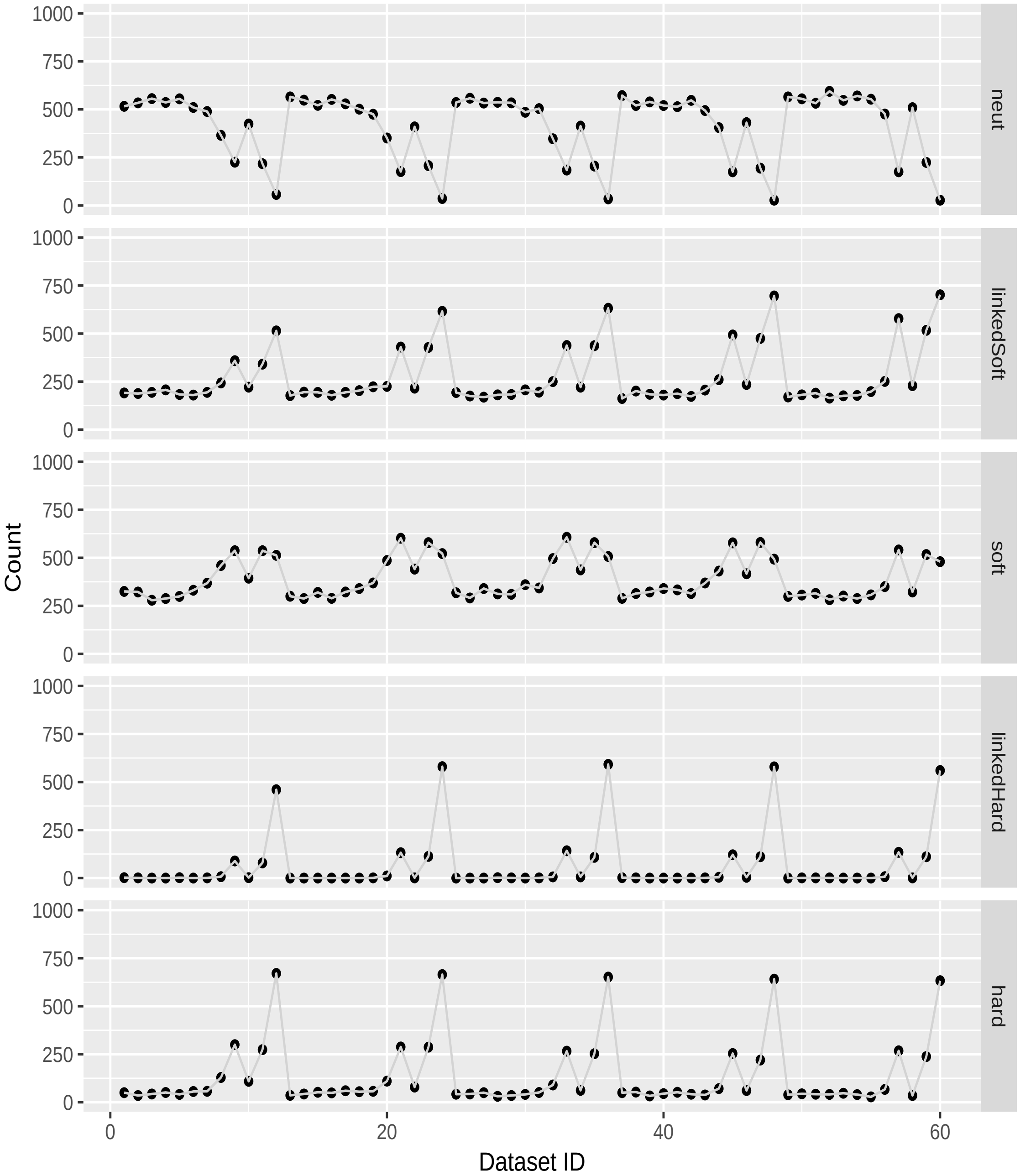

Supplementary Figure 2: **Classification of regions in the 60 neutral simulated datasets with bottlenecks (datasets 1–60) by S/HIC.** S/HIC classifies subgenomic regions as either hard sweep, soft sweep, linked-hard, linked- soft, or neutral. Linked-soft and linked-hard regions are neutral regions in the proximity (thus linked) of a soft and a hard selective sweep, respectively. As can be observed, S/HIC classified several regions in the 60 neutral simulated datasets as either soft sweeps, linked soft, hard sweeps, or linked hard, even though no selective sweeps were present.

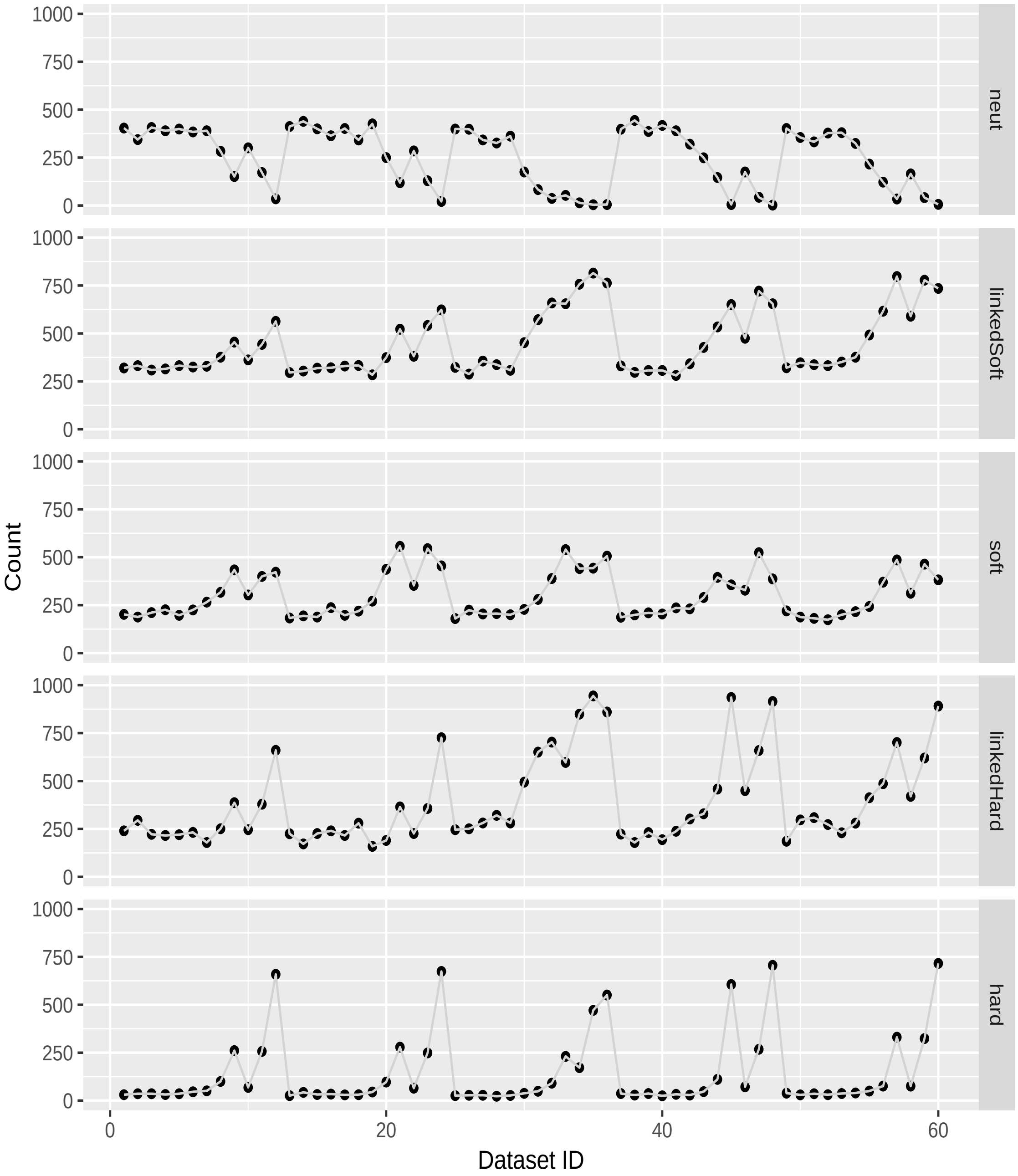

Supplementary Figure 3: **Classification of regions in the 60 simulated datasets with bottlenecks and a hard selective sweep (datasets 1–60) by S/HIC.** S/HIC classifies subgenomic regions as either hard sweep, soft sweep, linked- hard, linked-soft, or neutral. Linked-soft and linked-hard regions are neutral regions in the proximity (thus linked) of a soft and a hard selective sweep, respectively. As can be observed, S/HIC classified several regions in the 60 simulated datasets as either soft selective sweeps or linked soft, even though no soft selective sweeps were present.

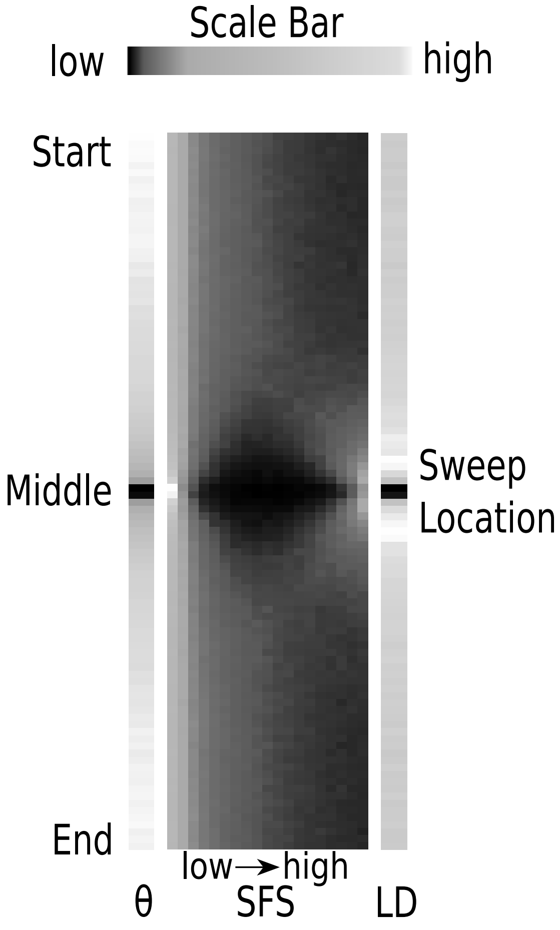

Supplementary Figure 4: **The three signatures of a selective sweep.** The figure illustrates the genetic variation (measured by Watterson's θ_W [1]), the shift of the SFS, and the emergence of LD patterns (measured by Wall's B statistic [2]) in the neighborhood of a selective sweep. The statistic values are averaged over 1,000 simulations with a selective sweep at the center of a 1-Mb genomic region. Simulations were conducted using the software mssel (kindly provided by R.R. Hudson), with $theta = 2,000$, $rho = 2,000$, and 2,000 recombination breakpoints. The population experienced a very mild bottleneck (0.5 of the present-day population size) at time 0.1 and recovered to the present-day size at time 0.1004. The figure shows that i) the average value of θ_W decreases near the sweep location, ii) the SFS obtains a U-shape pattern, with the number of low- and high-frequency derived variants elevated in comparison with the middle-frequency ones, and iii) the levels of LD increase locally in the two neighboring regions flanking the sweep location but not between them. The different shades of gray indicate low and high values, as described by the scale bar. In the leftmost heatmap, θ represents the expected (average) diversity, measured by Watterson's θ_W. The panel in the middle shows the SFS as it shifts from low-frequency derived variants to high-frequency ones. The rightmost heatmap shows the LD pattern, measured by Wall's B statistic, i.e., the number of congruent consecutive SNPs.

Supplementary Table 1

Seqnames	start	end	width	strand	gene_id	symbol
chr5	69812079	70585523	773445	-	100049076	GUSBP9
chr16	22503062	22547841	44780	+	100132247	NPIPB5
chr15	82647286	83084729	437444	+	100133144	NA
chr15	82647286	83084341	437056	+	100134869	UBE2Q2P2
chr16	21415198	21531765	116568	-	100271836	SMG1P3
chr1	144146811	146467744	2320934	+	100288142	NBPF20
chr8	86566828	86757761	190934	-	100288527	REXO1L2P
chr15	82821161	83209208	388048	-	100505503	NA
chr16	29465871	30215650	749780	+	100526831	SLX1B-SULT1A4
chr5	69422177	69881549	459373	-	11039	SMA4
chr5	69497639	69881549	383911	-	11042	SMA5
chr3	66119285	66438532	319248	+	115286	SLC25A26
chr7	153584419	154685995	1101577	+	1804	DPP6
chr20	34894303	35157040	262738	+	22839	DLGAP4
chr16	21413455	21458484	45030	-	23117	NPIPB3
chr22	21771693	21805750	34058	+	23119	HIC2
chr8	86568695	86840171	271477	-	254958	REXO1L1P
chr1	148003642	148346929	343288	-	25832	NBPF14
chr15	30488239	30665668	177430	+	26082	DKFZP434L187
chr7	130146080	130353598	207519	-	26958	COPG2
chr16	18511182	18573434	62253	-	283820	NOMO2
chr9	42844370	67032072	24187703	-	286297	LOC286297
chr7	74601106	74867341	266236	-	2970	GTF2IP1
chr15	29131168	29410516	279349	+	321	APBA2
chr22	21827287	21871780	44494	-	375133	PI4KAP2
chr17	34538468	34641846	103379	+	388372	CCL4L1
chr1	147835127	148176401	341275	-	388685	LINC01138
chr7	74379083	74438803	59721	+	389523	NA
chr15	82585621	82924242	338622	+	390660	ADAMTS7P1
chr19	197016	202209	5194	-	399844	LINC01002
chr9	39443818	41592207	2148390	-	401509	ZNF658B
chr17	34522268	34625716	103449	-	414062	CCL3L3
chr15	82711895	83108111	396217	+	440295	GOLGA6L9
chr16	29454226	30282198	827973	-	440354	SMG1P2
chr12	132680917	132905905	224989	-	50614	GALNT9
chr8	145192672	145440828	248157	+	51236	HGH1
chr16	29465822	30208887	743066	+	548593	SLX1A
chr16	29454226	30205627	751402	-	552900	BOLA2
chr10	47894023	51893269	3999247	+	55747	NA
chr13	114321597	114438637	117041	+	6011	GRK1
chr16	29460666	30200575	739910	+	606724	LOC606724
chr16	29476289	30218248	741960	-	613038	LOC613038
chr17	34522268	34625730	103463	-	6349	CCL3L1
chr16	22448329	22503541	55213	+	641298	SMG1P1
chr5	138778005	138842320	64316	-	641700	ECSCR
chr9	39355699	39891210	535512	+	642265	NA
chr15	84868830	85748518	879689	-	642423	LOC642423
chr8	145321517	145492131	170615	+	642658	SCX
chr16	14805546	14859315	53770	+	642778	NPIPA3
chr15	82633123	83018198	385076	-	647042	GOLGA6L10
chr5	68921201	69586004	664804	-	653188	GUSBP3
chr9	39443814	41609544	2165731	-	653501	NA

chr1	144676437	145039992	363556	-	653513	LOC653513
chr17	34581085	34808103	227019	-	654341	NA
chr5	69345350	70247953	902604	+	6606	SMN1
chr5	69345350	70248842	903493	+	6607	SMN2
chr16	29471207	29476301	5095	+	6818	SULT1A3
chr10	51224681	51371331	146651	-	728404	NA
chr10	51253908	51371316	117409	-	728407	PARGP1
chr10	48844036	49383240	539205	-	728798	FRMPD2B
chr1	144300512	144521969	221458	-	728875	NA
chr7	74807605	74867341	59737	-	729438	GATSL2
chr17	34745936	34806015	60080	-	729877	TBC1D3H
chr9	73149966	74061820	911855	-	80036	TRPM3
chr5	69321072	70214357	893286	+	8293	SERF1A
chr10	51026325	51729967	703643	-	8505	PARG
chr15	30653443	30685864	32422	-	89832	CHRFAM7A
chr1	144676437	145076186	399750	-	9659	PDE4DIP
chr1	17066768	17299474	232707	+	9696	CROCC

Short list of 60 genes with the highest RAiSD scores (top 0.05%), based on the analysis of the whole set of human autosomes (1000 Genomes data).

Supplementary Note 1

Current detection methods, such as SweepFinder [3], SweepFinder2 [4], SweeD [5], and OmegaPlus [6], require several input parameters. Some of these parameters (other parameters simply affect the format of the generated output files) determine how exhaustively each tool is going to scan a dataset. This is the case for the input parameter "-s" in SweepFinder and SweepFinder2, for instance, which allows the user to provide the number of genomic locations to evaluate the CLR test. Identical functionality provides the "-grid" parameter in SweeD and OmegaPlus, with the former evaluating the CLR test [3] while the latter calculating the ω *statistic* [7]. The aforementioned implementations construct a grid of equidistant locations to evaluate, based on the locations of the first and the last SNPs in the input data. This approach has implications on the accuracy of the detection process, as well as on the computational efficiency of the applied methods. This is due to the fact that the user's choice of the grid size and the location of the first and the last SNPs may lead to execution scenarios where no grid point is placed in the region of a selected locus. Without any candidate location to test for selection near a selected locus, the detection process will fail to accurately localize the selection target regardless of the implemented method. In addition, the placement of grid points along a genome is based on the size of the evaluated genomic region in base-pairs (bp), e.g., a grid point per kb. Given that only polymorphic sites are informative for the detection process, a bp-based creation of the evaluation grid may lead to redundant calculations in regions with a reduced number of SNPs. The requirement for input parameters inevitably turns the analysis to a function of the user-provided values, yielding highly probable that multiple runs of the same software processing the same dataset can lead to different outcomes, and thus different biological conclusions. The recently released software SweepFinder2[4] provides an alternative approach to the "-s" parameter by allowing the user to provide an additional input file with the locations of interest for the calculation of the CLR test. Nevertheless, the aforementioned problem remains, since the CLR locations are still determined by the user. An arbitrary choice for the grid size directs the tools to place the nearest CLR location to the selection target far from the actual point of interest in roughly half of the cases. Expectedly, increasing the grid size lowers the chances of missing the selection target, which, nevertheless, leads to considerably longer execution times.

Supplementary Discussion

The following report should not be considered as an effort to provide validity to our results simply because they make sense.

Beleza et al. [8] reported that the *APBA2* gene (located on chromosome 15, region: 29,131,168 - 29,410,516) is significantly associated with skin color (p value: 1.5×10^{-8}). The gene *APBA2*, along with *SLC24A5*, *TYR*, and *SLC24A2*, account for 35% of the total variance for the skin color. The same study reported that the CMS score for *APBA2* is significantly higher than expected, indicating that *APBA2* has evolved under strong positive selection. Other genes reported by this study [8] to affect the skin or eye color have not been found in our list of the top 0.05% genes.

Bradley and Benner[9] performed a phylogenomics analysis to gain insight into the function of a gene family of low copy repeats (LCRs) that contains the sulfotransferase (*SULT*) genes which are involved in drug metabolism, cancer, and hormone regulation. The study presented a model of expansion of this family in the hominoid lineage, a member of which is the *SULT1A3* gene. Positively selected protein sites that might have been central in adapting the SULT1A3 enzyme were identified using K_a/K_s, the ratio of non-synonymous to synonymous substitutions. The study suggested that the adaptive nucleotide substitutions control the substrate specificity [9]. Another locus that RAiSD reports as an outlier is the *DKFZP434L187* gene. Using the F_{ST}-based β statistic, Storz et al. [10] reported evidence of positive selection in populations *outside* Africa for this locus. They conducted a multilocus scan of microsatellite variability to identify regions of the human genome subject to continent-specific hitchhiking events. In contrast, we found evidence of positive selection in YRI, which is an African population, using, however, additional signatures (i.e., SFS- and LD-based ones) instead of only the local reduction of genomic diversity.

The aforementioned three genes are among the top 0.05% of RAiSD results over the entire genome. The *DUFFY* locus (also known as *DARC* or *ACKR1*), a canonical example of positive selection in humans [11, 12, 13, 14], is located in chromosome 1 (region 159,173,803–159,176,290 in hg19) and encodes a chemokine receptor that plays a major role in the infection of red blood cells by *Plasmodium vivax*, a causative agent for malaria. RAiSD evaluated two positions within

the *DUFFY* locus, 159,174,112 and 159,174,898, with p-values 0.01217 and 0.0036, respectively, compared to the rest of the evaluated positions in chromosome 1. With respect to the whole genome, the Duffy locus is among the top 5% of the results. SweeD, SweepFinder2, and OmegaPlus did not evaluate any position inside the Duffy locus due to the used grid size (10,000). The closest OmegaPlus position was 159,167,328, with a p-value of 0.022, whereas both SweeD's and SweepFinder2's closest position was 159,184,722, with p-values of 0.2136 and 0.2245, respectively. Note that, despite SweeD, SweepFinder2, and OmegaPlus using the same grid size, the evaluated positions were not identical due to numerical accuracy differences in the calculation of the decimal representation for the locations.

Supplementary References

[1] Watterson, G. On the number of segregating sites in genetical models without recombination. *Theor. Popul. Biol.* **7**, 256–276 (1975).

[2] Wall, J. D. Recombination and the power of statistical tests of neutrality. *Genet. Res.* **74**, 65–79 (1999).

[3] Nielsen, R. *et al.* Genomic scans for selective sweeps using snp data. *Genome Res.* **15**, 1566–1575 (2005).

[4] DeGiorgio, M., Huber, C. D., Hubisz, M. J., Hellmann, I. & Nielsen, R. Sweepfinder2: increased sensitivity, robustness and flexibility. *Bioinformatics* **32**, 1895–1897 (2016).

[5] Pavlidis, P., Živković, D., Stamatakis, A. & Alachiotis, N. Sweed: likelihood-based detection of selective sweeps in thousands of genomes. *Mol. Biol. Evol.* **30**, 2224–2234 (2013).

[6] Alachiotis, N., Stamatakis, A. & Pavlidis, P. Omegaplus: a scalable tool for rapid detection of selective sweeps in whole-genome datasets. *Bioinformatics* **28**, 2274–2275 (2012).

[7] Kim, Y. & Nielsen, R. Linkage disequilibrium as a signature of selective sweeps. *Genetics* **167**, 1513–1524 (2004).

[8] Beleza, S. *et al.* Genetic architecture of skin and eye color in an african-european admixed population. *PLos Genet.* **9**, e1003372 (2013).

[9] Bradley, M. E. & Benner, S. A. Phylogenomic approaches to common problems encountered in the analysis of low copy repeats: The sulfotransferase 1a gene family example. *BMC Evol. Biol.* **5**, 22 (2005).

[10] Storz, J. F., Payseur, B. A. & Nachman, M. W. Genome scans of dna variability in humans reveal evidence for selective sweeps outside of africa. *Mol. Biol. Evol.* **21**, 1800–1811 (2004).

[11] Sabeti, P. C. *et al.* Positive natural selection in the human lineage. *Science* **312**, 1614–20 (2006).

[12] Vallender, E. J. & Lahn, B. T. Positive selection on the human genome. *Hum. Mol. Genet.* **13**, R245–R254 (2004).

[13] Oleksyk, T. K., Smith, M. W. & O'Brien, S. J. Genome-wide scans for footprints of natural selection. *Philos. Trans. R. Soc. Lond., B, Biol. Sci.* **365**, 185–205 (2010).

[14] McManus, K. F. *et al.* Population genetic analysis of the darc locus (duffy) reveals adaptation from standing variation associated with malaria resistance in humans. *PLos Genet.* **13**, e1006560 (2017).
