# Supplementary material for: RAiSD detects positive selection based on multiple signatures of a selective sweep and SNP vectors
Source: Commun Biol. 2018 Jun 27;1:79. doi: 10.1038/s42003-018-0085-8 (PMC6123745; doi:10.1038/s42003-018-0085-8)
Supplement: Supplementary file 2 — Description of additional supplementary items [file 42003_2018_85_MOESM2_ESM.pdf]

Description of Additional Supplementary Files

File Name: Supplementary Data 1
Description: Command lines for dataset generation. Lines 1-113 contain command lines for BASE datasets, i.e., those used to calculate the cutoff value. Lines 114-226 contain command lines for TEST datasets, i.e., those used to test for selection and/or the effect of confounding factors. The trajectory files are available at: https://doi.org/10.6084/m9.figshare.6339533.
